# Characterization and isolation of highly purified porcine satellite cells

**DOI:** 10.1038/cddiscovery.2017.3

**Published:** 2017-04-10

**Authors:** Shijie Ding, Fei Wang, Yan Liu, Sheng Li, Guanghong Zhou, Ping Hu

**Affiliations:** 1 Key Laboratory of Meat Processing and Quality Control, College of Food Science and Technology, Nanjing Agricultural University, Nanjing 210095, China; 2 State Key Laboratory of Cell Biology, Center of Excellence in Molecular and Cell Biology, Shanghai Institute of Biochemistry and Cell Biology, Chinese Academy of Sciences, 320 Yueyang Road, Shanghai 200031, China; 3 University of Chinese Academy of Sciences, 320 Yueyang Road, Shanghai 200031, China

## Abstract

Pig is an important food source and an excellent system to model human diseases. Careful characterization of the swine skeletal muscle stem cells (satellite cells) will shed lights on generation of swine skeletal muscle disease model and efficient production of porcine meat for the food industry. Paired box protein 7 (Pax7) is a highly conserved transcription factor shared by satellite cells from various species. However, the sequence of Pax7 has not been characterized in pig. The lack of method to isolate highly purified satellite cells hinders the thorough characterization of the swine satellite cells. Here we found molecular markers for swine satellite cells and revealed that the porcine satellite cells were heterogeneous in various pieces of skeletal muscle. We further developed a method to isolate highly purified satellite cells directly from porcine muscles using fluorescence-activated cell sorting. We next characterized the proliferation and differentiation abilities of isolated satellite cells *in vitro*; and found that long-term culturing of satellite cells *in vitro* led to stemness loss.

## Introduction

Satellite cells are a heterogeneous population of adult stem cells located in skeletal muscles.^1–4^ These cells reside between the muscle sarcolemma and the basal lamina of muscle fibers and remain quiescent under normal conditions.^1,2,5,6^ Upon injury, quiescent satellite cells are activated to repair muscle injury while a subset of the activated satellite cells return to quiescent status after self-renewal.^1,2,7^ Satellite cells are essential for muscle regeneration and mice lacking satellite cells display severe muscle regeneration defects.^8–10^ Satellite cells impairment occurs in muscular diseases such as Duchenne muscular dystrophy (DMD)^11^ and in aged muscles.^12^

Pigs have many advantages in modeling human diseases due to their similar anatomic and physiological features to human beings.^13,14^ For example, the swine DMD model recapitulates human symptoms better than mouse model. The severe progressive dystrophic changes of skeletal muscles, impaired mobility, muscle weakness, and a much shorter life span are common symptoms in human DMD patients.^15^ These symptoms can only be recapitulated in pig DMD model,^16^ but not mouse DMD model,^17^ supporting the notion that pigs can model human diseases better than rodents. Characterization of satellite cells in pig will be a valuable addition to our understanding of the porcine model system.

Paired box protein 7 (Pax7) has been shown to be the critical regulator of satellite cell maintenance and proliferation in several species.^18–20^ Pax7 is a highly conserved protein present in many mammalian species such as human, mouse, and cattle. In mouse, Pax7 can directly regulate MyoD and Myf5 to modulate satellite cell maintenance and proliferation.^21–24^ The full-length CDS of Pax7 has been found in mouse (Accession #: NM_011039) and human (Accession #: NM_001135254) database. But there are only three short partial sequences for *Pax7* in pig existing in the database currently.

Satellite cells frequency and functions are heterogeneous in both mouse and human skeletal muscles.^4,25,26^ Mouse soleus and diaphragm muscles have more satellite cells per mm^3^ compared to other limb muscles.^25^ Human temporalis muscle has estimated higher satellite cell while other body and limb muscles being analyzed show similar satellite cell numbers.^26^ But little is known about the satellite cell frequency heterogeneity in pig muscles.

Highly purified mouse satellite cell population can be obtained by fluorescence-activated cell sorting (FACS) sorting. But the method to purify porcine satellite cells is not well established. Several approaches have been reported to isolate the myogenic lineage cells from porcine muscle tissues, including Percoll gradient centrifugation and preplating.^27–31^ However, the cell identity of the myogenic lineage population obtained by the above methods has not been carefully characterized yet. The purification efficiency of Percoll gradient centrifugation has big variation as reported by different groups.^27,32^ Furthermore, in cells obtained by Percoll gradient centrifugation, only a portion (<60%) of them stain positive for neural cell adhesion molecule (N-Cam), which is considered to be a porcine satellite cell marker.^28^ Consistently, only 65% of the cells obtained by this method are able to fuse and form myotubes.^33^ Another widely used approach to isolate porcine myogenic lineage cells is the preplating method.^29,34,35^ Pax7 immunofluorescent staining indicates that satellite cells only account 20–50% of the cell population obtained by this method.^29,35^ Thus, it is necessary to develop a more efficient method to obtain highly purified satellite cells.

Previous studies in mouse have suggested that highly purified satellite cells can be obtained by FACS sorting.^2,36,37^ The cell surface markers for FACS sorting have been well characterized in murine muscle tissues. The most commonly used cell surface markers in murine are CD34 and *α*7-integrin.^2,36,38^ The cell surface markers to specify satellite cells in pig are not well characterized. It has been reported that CD56 was expressed in a myogenic cell population residing in human^26,39^ and swine skeletal muscles.^40^ Further analysis in human muscles showed that the cells expressing CD56 can be further divided to two populations based on CD34 expression. CD56^+^CD34^−^ cells isolated from skeletal muscles displayed sole myogenic potency; while CD56^+^CD34^+^ cells showed both adipogenic and myogenic potentials,^40^ though the scenario in pig has not been well characterized. CD29 (*β*1-integrin) is one of the integrin family members that can form heterodimers to link the extracellular matrix to actin cytoskeleton within cells.^41^ CD29-deficient embryonic myoblasts displayed impaired fusion ability *in vitro*.^42^ Satellite cells lacking CD29 fail to maintain quiescent stage and cannot sustain the expansion and self-renewal of this stem cell pool during regeneration.^43^ In mouse, more than 95% of satellite cells are CD29 and Pax7 double positive;^44^ in human, 96% of satellite cells are CD29 and Pax7 double positive.^26^ These observations suggest that CD29 could be a surface marker to specify satellite cells together with other markers.

It has been reported that long-term cultivation of satellite cells *in vitro* resulted in loss of most of the regenerative potentials *in vivo* after transplantation in mouse,^36,45^ canine,^46^ and human.^47^ Scattered reports have shown that the progenitor cells derived from swine skeletal muscles display multilineage differentiation capacities after being cultured *in vitro* for 3 months,^48^ but the identity of these cells has not been characterized. Whether the lack of ability to be expanded *in vitro* is also true in pig satellite cells is not known yet.

Here we characterize the satellite cell frequency in variant pieces of porcine skeletal muscles. The cell surface markers specific for porcine satellite cells have been identified, and a FACS sorting method to isolate porcine satellite cells based on the surface markers has been established. We further demonstrate that similar to satellite cells in other species, porcine satellite cells lose their stem cell identities and differentiation potentials after long-term *in vitro* culturing.

## Results

### Clone of the full-length porcine *Pax7* gene

**Figure 1:**
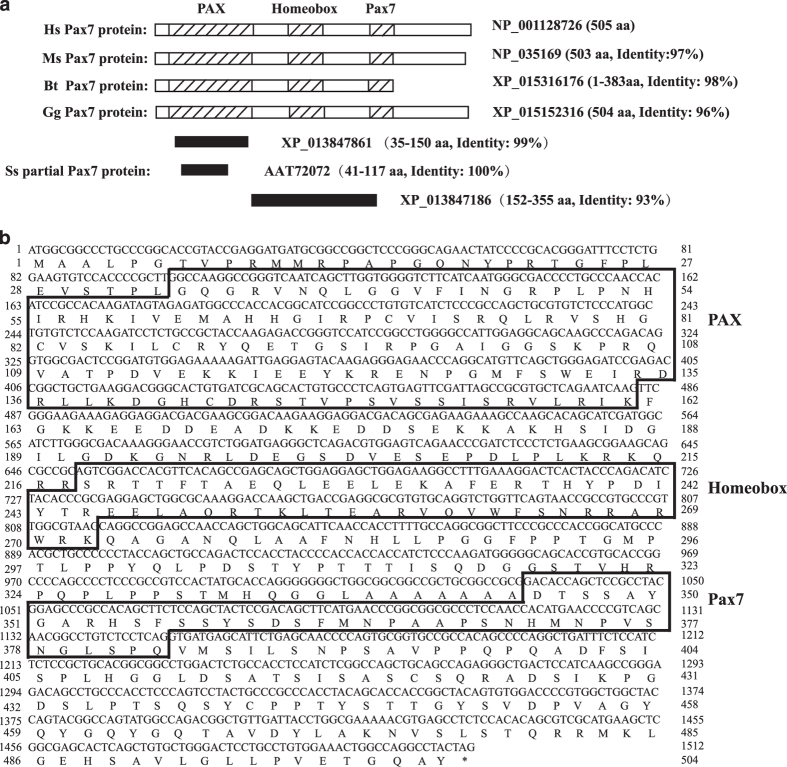
Cloning of the full-length porcine *Pax7* CDS. (**a**) Schematic representation of the Pax7 protein sequences from different species. Shadow indicated the conserved domains. Mouse (*Mus musculus*, Ms), cattle (*Bos taurus*, Bt), chick (*Gallus gallus,* Gg) Pax7 proteins, and partial pig (*Sus scrofa*, Ss) Pax7 peptide sequences were aligned with human (*Homo sapiens*, Hs) Pax7 protein. Black indicated the partial porcine *Pax7* cDNA sequences. (**b**) The nucleotide sequence of pig *Pax7* and the predicted protein sequence. The conserved PAX, Homeobox, and Pax7 were boxed.

Pax7 is an important regulator of muscle satellite cells.^18–20^ We set out to compare the similarity of Pax7 peptide sequence between pig and other species. However, we could not find the full-length Pax7 sequence in the NCBI database. There are only several partial porcine *Pax7* cDNA sequences in NCBI nucleotide database (Accession #: AY653213, XM_013992407, and XM_013991732). These translated peptide sequences were aligned with Pax7 peptide sequences from several species (Figure 1a). Peptide sequence (Accession #: AAT72072) translated from AY653213 displayed 100% identity to Pax7 peptide sequences from multiple species (Figure 1a and Supplementary Figure S1). Pax3 belongs to the same family as Pax7 and share high similarity to Pax7. Sequence comparison indicated differences on two key amino acids between peptide sequence derived from AY653213 and Pax3 amino-acid sequence from various species (Supplementary Figure S1), suggesting that AY653213 could be a partial sequence from porcine *Pax7*.

To identify the complete cDNA sequence of *Pax7* in pig, we performed 5′ and 3′ rapid amplification of cDNA ends (RACE) using AY653213 as the seed sequence with total RNA extracted from porcine myoblasts (Supplementary Figure S2a). A 400 bp fragment was generated by the 5′ RACE; and a 5000 bp fragment was generated by the 3′ RACE (Supplementary Figure S2b). The two fragments could be assembled into a 1512 bp CDS (Accession #: KX815346), which encoded a 503 amino-acid polypeptide (Figure 1b). The assembled nucleic acid sequence showed 93% identity to human *Pax7* and 90% identity to mouse Pax7 (Supplementary Figure S3a). The translated polypeptide displayed 99% identity to human Pax7 and 98% identity to mouse Pax7 protein (Supplementary Figure S3b). Paired box domain (PAX), homeobox domain (Homeobox), and Pax7 domain, the three highly conserved domains of Pax7 from various species, were all found in the amino-acid sequence translated from the full-length pig *Pax7* CDS (Figure 1b).

### The satellite cell number varied at different age and location

**Figure 2:**
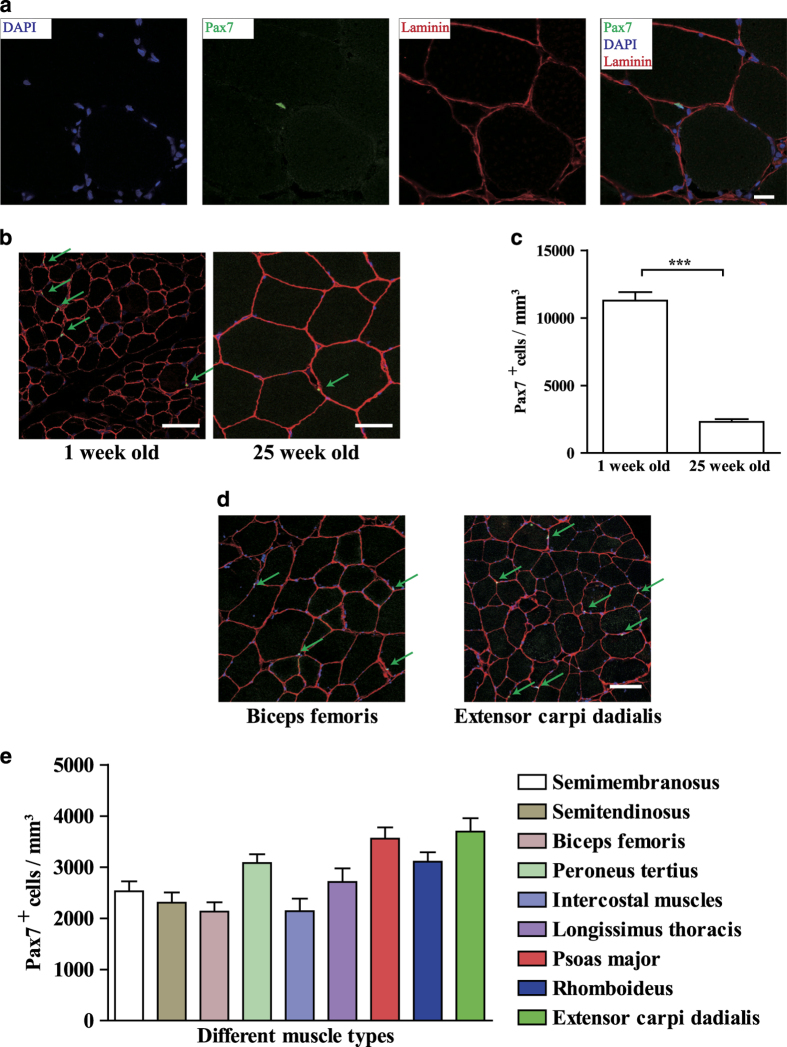
The number of satellite cells varied at different age and location. (**a**) Immunofluorescent staining of the semitendinosus muscle section from 25-week-old pig using Pax7 and Laminin antibodies. Green indicated Pax7; red indicated Laminin; blue indicated DAPI. Scale bars, 20 *μ*m. (**b**) Representative images of immunofluorescent staining of Pax7 (satellite cells) and Laminin on pig semitendinosus cross sections obtained from 1-week or 25-week-old pigs. Green indicated Pax7; red indicated Laminin; blue indicated DAPI. The green arrows indicated the satellite cells. Scale bars, 50 *μ*m. (**c**) Quantifications of the satellite cell numbers in mice at different ages. Error bars represented S.E.M. and were based on four independent experiments. Significance was analyzed by student’s *t*-test, *** indicated *P*<0.001. (**d**) Representative images of immunofluorescent staining of pig satellite cells on tissue sections derived from different muscle types in 25-week-old pigs. Green indicated Pax7; red indicated Laminin; blue indicated DAPI. The green arrows indicated the satellite cells. Scale bars, 100 *μ*m. (**e**) Quantifications of satellite cell number in different pieces of skeletal muscles. Error bars represented S.E.M. and were based on four independent experiments.

We tested several antibodies and found the antibody recognizing porcine Pax7 protein. This antibody was utilized to detect the endogenous porcine satellite cells. Consistent with observations in mouse^18^ and human,^26^ Pax7-positive satellite cells were detected underneath the basal membrane as indicated by Laminin immunofluorescent staining in both postnatal and adult pigs (Figures 2a and b).

The satellite cell number is important for skeletal muscle homeostasis maintenance. It has been reported that the satellite cell number decreased in adult when compared to neonatal mice.^49^ The number of satellite cells also differed between various pieces of skeletal muscles in the same individual.^25^ We next checked the satellite cell number in semitendinosus from 1- and 25-week-old pig by Pax7 immunofluorescent staining. The number of satellite cells decreased in adult skeletal muscle (25 week old) compared to neonatal (1 week old) muscles in pig (Figures 2b and c). To compare the satellite cell numbers in different muscle pieces, we analyzed nine types of adult pig skeletal muscle from different locations. Peroneus tertius, psoas major, and extensor carpi radialis had high number of satellite cells; while intercopstal muscle and biceps femoris had low amount of satellite cells (Figures 2d and e). These results suggest that the distribution of satellite cells in different type of skeletal muscle is different.

### Identification of the satellite cell-specific cell surface markers

**Figure 3:**
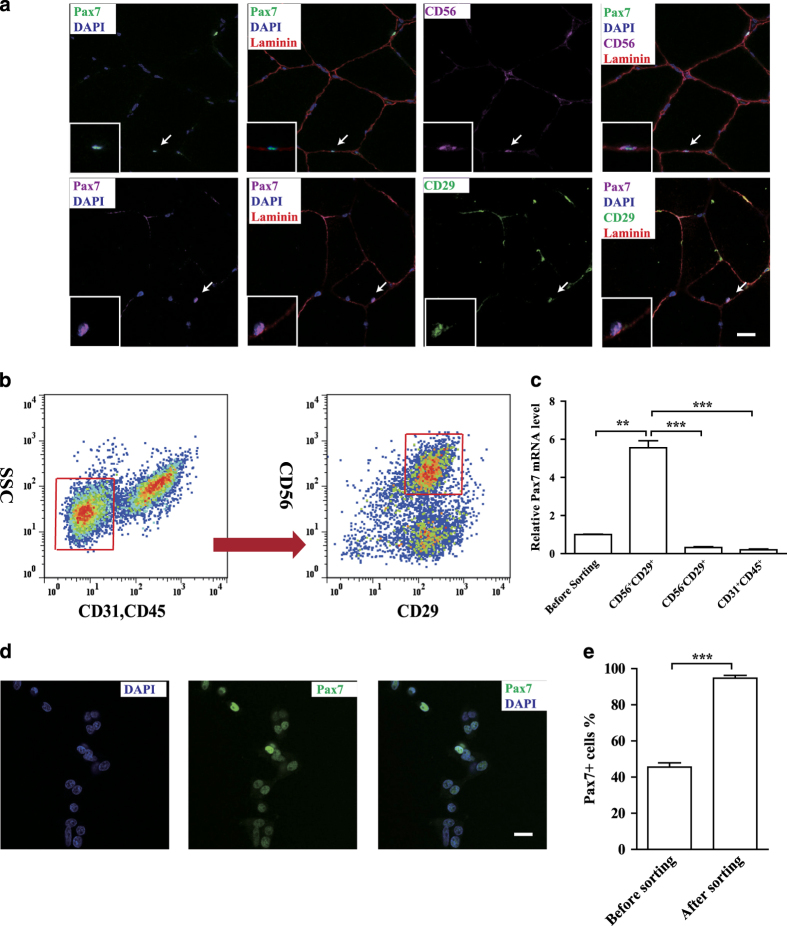
Identification of the satellite cell-specific cell surface markers. (**a**) Immunofluorescent staining of Pax7, CD56 (**a**, top), CD29 (**a**, bottom) and Laminin on cross sections of semitendinosus muscle from 25-week-old pig. Green indicated Pax7 (**a**, top) or CD29 (**a**, bottom); purple indicated CD56 (**a**, top) or Pax7 (**a**, bottom); blue indicated DAPI; red indicated Laminin. Arrows indicated satellite cells expressing all three markers. Scale bars, 20 *μ*m. (**b**) FACS analysis of mononuclear cells from semitendinosus obtained from 1-week-old pig. Cells were gated by forward scatter and side scatter (not shown) prior to gating for CD45/31, CD56, and CD29. Red gates indicated sub-populations containing pig satellite cells. (**c**) qRT-PCR analysis of Pax7 mRNA levels in freshly isolated pig satellite cells and other cell populations were shown. Error bars represented S.E.M. and were based on four independent experiments. (**d**) Immunofluorescent staining of Pax7 in pig satellite cells cultured 4 days *in vitro*. Blue indicated DAPI; green indicated Pax7. Scale bars, 20 *μ*m. (**e**) Quantification of Pax7 immunofluorescent staining of sorted CD56^+^CD29^+^ cells and unsorted cells cultured 4 days *in vitro* from 1-week-old pig. Error bars represented S.E.M. and were based on three independent experiments. Significance was analyzed by student’s *t*-test, ** indicated *P*<0.01, *** indicated *P*<0.001.

In order to set up FACS sorting method to isolate porcine satellite cells, we next characterized the specificity of the surface marker for porcine satellite cells. CD56 and CD29 have been reported to mark satellite cells in human. We performed immunofluorescent staining assays to verify whether these surface markers were also applicable to porcine satellite cells. Both CD56 and CD29 co-localized in the same cell with Pax7 in porcine skeletal muscles (Figure 3a), suggesting that they could be used as cell surface marker for isolation of satellite cells from pig skeletal muscles. Antibodies against CD31 (an endothelial cell marker)^50^ and CD45 (a hematopoietic cell marker)^48^ were utilized to distinguish the endothelial and hematopoietic cells present in the mononuclear cells isolated from porcine skeletal muscles. Based on these results, we isolated CD31^−^CD45^−^CD56^+^CD29^+^ cells from mononuclear cell population obtained from porcine skeletal muscles by FACS sorting (Figure 3b). The *Pax7* and *Myf5* mRNA level in this cell population was compared with that in CD31^+^CD45^+^ and CD31^−^CD45^−^CD56^−^CD29^+^ populations by RT-qPCR. *Pax7* and *Myf5* are enriched in CD31^−^CD45^−^CD56^+^CD29^+^ population (Figure 3c and Supplementary Figure S4a). Immunofluorescent staining assays revealed that 94% of CD31^−^CD45^−^CD56^+^CD29^+^ cells express Pax7 (Figures 3d and e and Supplementary Figure S4b), suggesting that the specific enrichment of Pax7^+^ cells in CD31^−^CD45^−^CD56^+^CD29^+^ cell population isolated from porcine skeletal muscles. Taken together, satellite cells can be isolated from pig skeletal muscles by FACS sorting using CD31 and CD45 negative selection followed by CD56 and CD29 positive selection.

**Figure 4:**
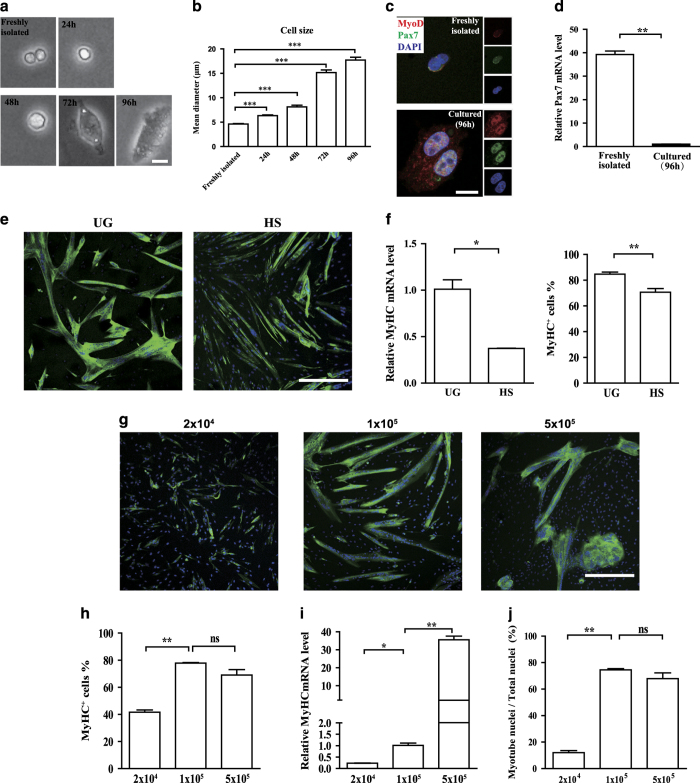
Proliferation and differentiation abilities of pig satellite cells *in vitro*. (**a**) Representative phase contrast images of pig satellite cells cultured in F-10 medium for various hours. Scale bars, 10 *μ*m. (**b**) Quantification of the cell size with different culturing time. Error bars represented S.E.M. and were based on three independent experiments. (**c**) Representative immunofluorescent staining images of pig freshly isolated satellite cells or cultured *in vitro* for 96 h. In the insets, red indicated MyoD; green indicated Pax7; blue indicated DAPI. Scale bars, 10 *μ*m. (**d**) qRT-PCR analysis of *Pax7* mRNA levels in freshly isolated pig satellite cells and satellite cells cultured for 96 h. Error bars represented S.E.M. and were based on three independent experiments. (**e**) Representative immunofluorescent staining images of MyHC of differentiated satellite cells at passage 1 with 0.4% UG and 2% HS. Green indicated MyHC; blue indicated DAPI. Scale bars, 50 *μ*m. (**f**) qRT-PCR analysis of *MyHC* mRNA levels with cells differentiated in 0.4% UG and 2% HS conditions (**f**, left). Error bars represented S.E.M. and were based on three independent experiments. Quantification of the number of MyHC^+^ cell numbers with cells differentiated in 0.4% UG and 2% HS conditions (**f**, right). Error bars represented S.E.M. and were based on three independent experiments. (**g**) Representative immunofluorescent staining images of MyHC with different amount of seed cells at passage 3. Cells were cultured with 2×10^4^ (left), 1×10^5^ (middle), 5×10^5^ (right) cells for 2 days, then induced to differentiate in 0.4% UG. Green indicated MyHC; blue indicated DAPI. Scale bars, 50 *μ*m. (**h**) Quantification of the number of MyHC^+^ cells in cells differentiated from different amount of seed cells, error bars represented S.E.M. and were based on three independent experiments. (**i**) qRT-PCR analysis of *MyHC* mRNA levels in cells differentiated from different amount of seed cells. Error bars represented S.E.M. and were based on three independent experiments. (**j**) Quantification of the percentage of nuclei in myotubes differentiated from different amount of seed cells. Error bars represented S.E.M. and were based on three independent experiments. Significance was analyzed by student’s *t*-test. * indicated *P*<0.05, ** indicated *P*<0.01. *** indicated *P*<0.001. NS indicated no significant difference.

To analyze the proliferation abilities of porcine satellite cells *in vitro*, we cultured the satellite cells for several days *in vitro*. After being cultured *in vitro* for 96 h, the cell size started to increase and the cell shape became more spindle-like (Figures 4a–c and Supplementary Figure S5a). Previous reports have shown that the cell size increased when satellite cells being activated and differentiating to myoblasts.^51^ To further characterize the identity of the cells isolated from pig skeletal muscles by FACS sorting, we performed immunofluorescent staining of MyoD and Pax7. In the round satellite cells which were cultured *in vitro* for less than 6 h, Pax7 protein was detected. MyoD protein level was relatively low (Figure 4c). While in cells being cultured for 96 h with bigger cell size, Pax7 was also detected (Figure 4c). MyoD protein displayed punctuated distribution and higher level in nuclei (Figure 4c). The *Pax7* mRNA level was decreased during *in vitro* culturing (Figure 4d). These results suggest that consistent with the circumstance in mouse,^5,51^ the porcine satellite cells being cultured *in vitro* for over 96 h are activated and started to differentiate to myoblasts.

We next assayed the differentiation potentials of the porcine satellite cells *in vitro*. Porcine satellite cells were differentiated in either 2% horse serum (HS) or 0.4% Ultroser G (UG) (Figures 4e and f). The myosin heavy chain expression level was dramatically higher in UG-induced differentiation (Figure 4f). The differentiation efficiency was also enhanced by UG induction (Figure 4f).

We next examined whether cell density would affect pig satellite cell differentiation. We seeded 2×10^4^, 1×10^5^, and 5×10^5^ pig satellite cells in 3.5 cm dish for 2 days (Supplementary Figure S5b) and induced differentiation with UG, respectively. When the density of the seeded pig satellite cells was low, the differentiation efficiency decreased; when the cell density reached the threshold, the differentiation efficiency plateaued (Figures 4g and h). Higher seeding cell density yielded myotubes with elevated MyHC expression level and higher percentage of myotube nuclei (≥3 nuclei) in total nuclei (Figures 4i and j). Together, these results suggest that high seed cell density facilitates pig satellite cell differentiation.

We further examined the contractility of the differentiated myotubes. After being cultured in medium containing UG and Ca^2+^ for 6 days, myotubes differentiated from swine satellite cells were able to contract (video was included in Supplementary File S7). These results suggest that swine satellite cells were able to generate myotubes with contractility.

### Porcine satellite cells lost their stemness after long-term *in vitro* expansion

**Figure 5:**
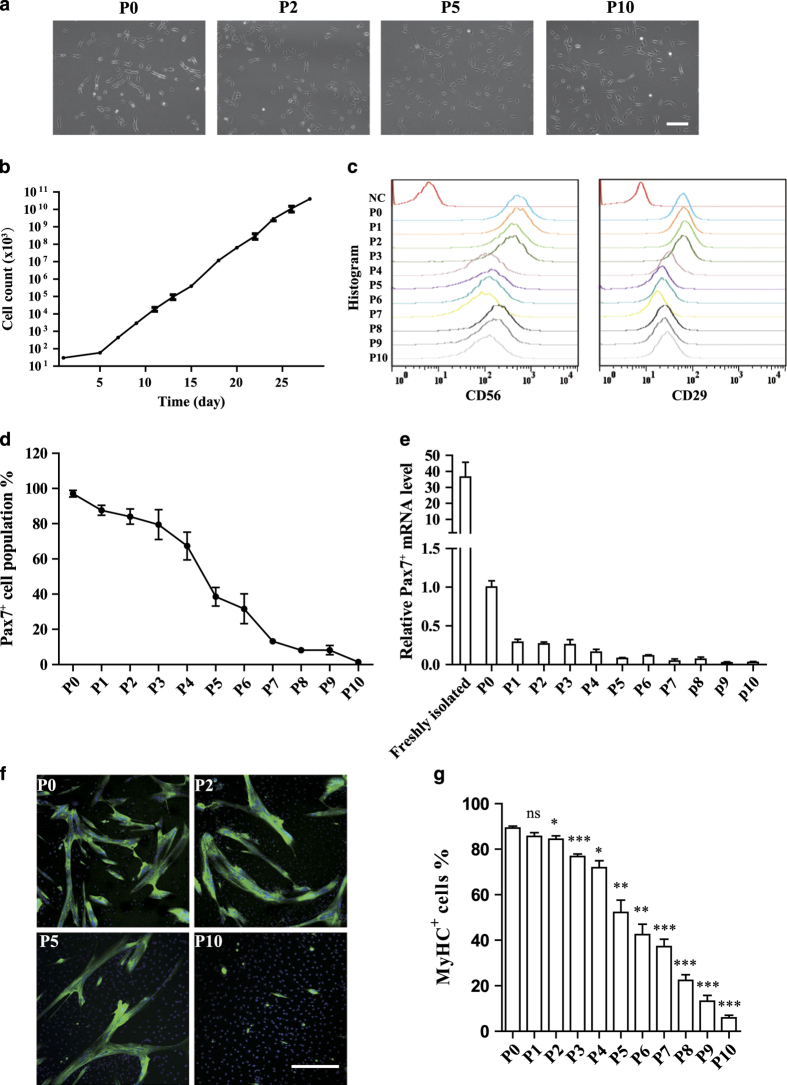
Porcine satellite cells lost their stemness after long-term expansion *in vitro*. (**a**) Representative phase contrast images of pig satellite cells cultured in F10 medium for different passages. Scale bars, 200 *μ*m. (**b**) Growth curves of pig satellite cells isolated from 1-week-old pig. Cells were serially expanded and counted at each passage. (**c**) FACS analysis of CD56 and CD29 expression from different passages of pig satellite cells cultured in F-10 medium. (**d**) Quantification of the number of Pax7^+^ cells from different passages. Error bars represented S.E.M. and were based on three independent experiments. (**e**) qRT-PCR analysis of *Pax7* mRNA levels from cells being expanded for different passages. Error bars represented S.E.M. and were based on three independent experiments. (**f**) Representative immunofluorescent staining images of MyHC staining of myotubes differentiated from P0, P2, P5, and P10 satellite cells. Green indicated MyHC; blue indicated DAPI. Scale bars, 50 *μ*m. (**g**) Quantification of MyHC^+^ cell numbers differentiated from satellite cells being serially expanded *in vitro*. Error bars represented S.E.M. and were based on three independent experiments. Significance was analyzed by student’s *t*-test. * indicated *P*<0.05, ** indicated *P*<0.01, *** indicated *P*<0.001. NS indicated no significant difference.

Both mouse and human satellite cells have been reported to suffer the loss of abilities to repair muscle injury *in vivo* after days of *in vitro* culturing.^5,36,45,47,52^ We therefore examined whether porcine satellite cells share the same feature. As described above, the cell size of porcine satellite cells increased and the cell shape became more spindle-like after 96 h of *in vitro* culturing (Figure 4a), suggesting these cells to be in a more differentiated status. The morphology of porcine satellite cells remained to be spindle-like after serial expansion *in vitro* (Figure 5a). These cells could be propagated logarithmly *in vitro* (Figure 5b). To further characterize the identity of the cells being serially expanded *in vitro*, CD56 and CD29 expression was analyzed by FACS. The expression level of both CD56 and CD29 decreased after passage 4 (Figure 5c), suggesting the gradual loss of satellite cell characters. Consistently, the percentage of Pax7^+^ cells also decreased dramatically after passage 4 and 5 (Figure 4d). The expression level of *Pax7* echoed the morphology changes observed above. After 96 h of *in vitro* culturing, the *Pax7* mRNA level dropped over 30 fold (Figure 5e). Serial expansion *in vitro* of these cells further drove the *Pax7* mRNA level and the percentage of Pax7^+^ cells down (Figures 5d and e). After passage 4 and 5, the *Pax7* expression level decreased to very low level (Figure 5e), suggesting that these cells have lost their satellite cell features. Consistently, the cells expanded *in vitro* for over 5 passages displayed significant lower differentiation potentials (Figures 5f and g). Taken together, these results suggest that pig satellite cells quickly lose their stem cell features *in vitro*.

**Figure 6:**
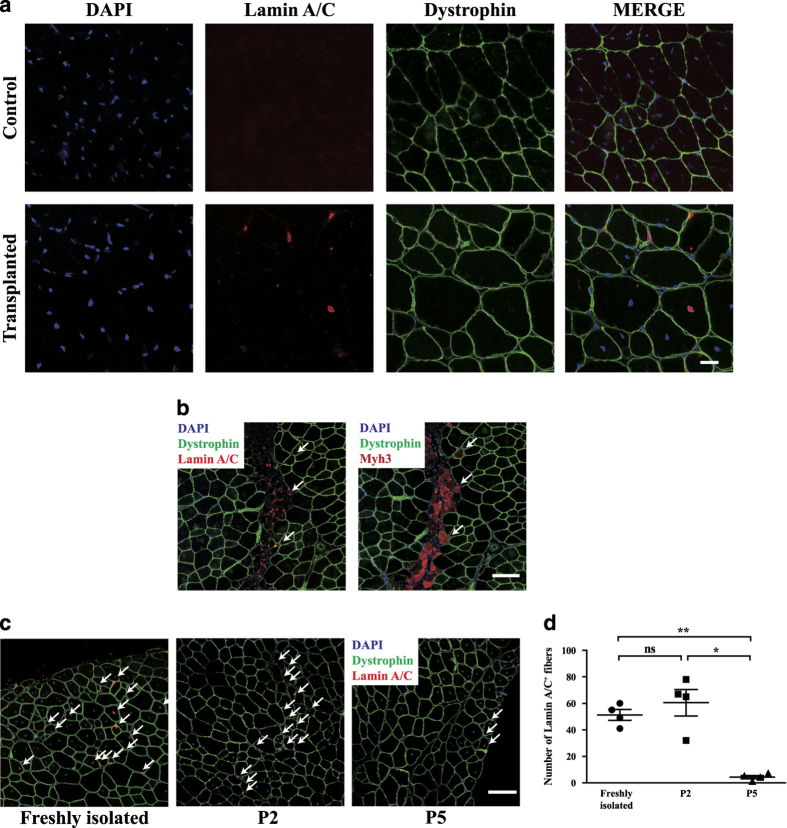
Pig satellite cells participated in the muscle regeneration in recipient mice and lose the engraftment efficiency after long-term expansion *in vitro*. (**a**) Four weeks after transplantation, engraftment efficiency of freshly isolated pig satellite cells was determined by Dystrophin and Lamin A/C immunofluorescent staining with muscle sections derived from receipient mice. PBS was injected as negative control. Blue indicated DAPI; red indicated Lamin A/C; green indicated Dystrophin. Scale bars, 20 *μ*m. (**b**) Lamin A/C, Myh3 immunofluorescent staining with series muscle sections derived from recipient mice 7 days after transplantation. Blue indicated DAPI; red indicated Lamin A/C (**b**, left) or Myh3 (**b**, right); green indicated Dystrophin. Scale bars, 100 *μ*m. (**c**) Representative immunofluorescent staining images of Dystrophin and Lamin A/C on mouse sections from mice injected with freshly isolated (**c**, left), P2 (**c**, middle), and P5 (**c**, right) satellite cells. Blue indicated DAPI; red indicated Lamin A/C; green indicated Dystrophin. Scale bars, 100 *μ*m. (**d**) Quantification of the number of Lamin A/C^+^ fibers in each TA 4 weeks after transplantation. Error bars represented S.E.M. and were based on four independent experiments. Significance was analyzed by student’s *t*-test. * indicated *P*<0.05, ** indicated *P*<0.01. NS indicated no significant difference.

We further examined the ability of pig satellite cells to repair muscle injury *in vivo* by cell transplantation. Satellite cells were isolated from 1-week-old piglets by FACS sorting. TA muscle injury in immunodeficient recipient mice (NOD.Cg-*Prkdc*^*scid*^ *Il2rg*^*tm1Wjl*^/SzJ) was induced by cardiotoxin injection. Various numbers of pig satellite cells were transplanted to the recipient mice. Four weeks after transplantation, TA muscles from recipient mice were collected to make cryo-sections and stained with antibody specifically recognizing pig Lamin A/C. Pig Lamin A/C can be detected in TA muscles of the recipient mice (Figure 6a and Supplementary Figure S6a). One week after transplantation, the porcine Lamin A/C could also be detected in newly formed myofibers (indicated by Myh3 staining; Figure 6b), suggesting that the transplanted pig satellite cells are able to engraft and contribute to muscle regeneration in recipient mice. We further examined whether the transplanted swine satellite cells were capable of homing to the right niche. Pax7-expressing satellite cells were detected in TA muscles of the recipient mice 4 weeks after transplantation (Supplementary Figure S6b). The Pax7-expressing satellite cells of pig origin (indicated by Lamin A/C staining) were detected at the similar location where the endogenous satellite cells resided (Supplementary Figure S6c). These results suggest that the transplanted swine satellite cells were capable of homing to the niche.

We next investigated whether *in vitro* expansion of porcine satellite cells affects their engraftment efficiency. The same number of freshly isolated pig satellite cells and porcine satellite cells expanded *in vitro* for different period were transplanted to immunodeficient recipient mice, respectively. The antibody specifically recognizing porcine Lamin A/C was used to perform immunofluorescent staining with muscle tissue sections 4 weeks after transplantation. The pig satellite cells expanded *in vitro* for 2 passages displayed similar engraftment efficiency with the freshly isolated pig satellite cells. However, few myofibers generated from pig satellite cells could be detected when these cells were expanded *in vitro* for 5 passages (Figures 6c and d), suggesting that pig satellite cells lost their stemness and abilities to repair muscle injury *in vivo* after prolonged *in vitro* expansion. These results are also consistent with the morphology and differentiation potential changes described above (Figures 4 and 5), suggesting that pig satellite cells lost their stem cell features after being expanded *in vitro* for 5 passages. Taken together, these results suggest that pig satellite cells lost their stemness and differentiation abilities after *in vitro* expansion, a feature shared by both mouse and human satellite cells.

## Discussion

Here we characterized pig muscle satellite cells and provided basic information for further utilization of these cells. We cloned the full-length cDNA sequence of porcine *Pax7* and characterized the numbers of satellite cells located at various piece of skeletal muscles. A FACS-sorting-based method was established to isolate highly purified pig satellite cells. Using this method, we isolated pig satellite cells and found that they lost their stem cell morphology, differentiation potential, and ability to repair muscle injury *in vivo* after prolonged *in vitro* culturing.

The neonatal mice have high numbers of satellite cells, while the number decreased in the adult (reviewed by Fu *et al.*).^53^ Similar phenomenon has been observed in pig. The number of satellite cells was much higher in 1-week-old pig compared to that in 25-week-old pig. The number of satellite cells varied at different locations. In mouse, extraocular and diaphragm have high number of satellite cells, Gastrocnemius and TA have low number of satellite cells.^25^ In human muscles, it is indicated that under normal homeostatic conditions, most adult human skeletal muscle contains a relatively homogeneous frequency of Pax7 satellite cells per fiber.^26^ Both pig and human have larger fibers than mouse, but the relative homogeneous frequency of satellite cells in pig fibers still needs more studies.

The loss of abilities to repair muscle injury *in vivo* of satellite cells after being cultured *in vitro* (empty amplification) is a common problem shared by mouse and human satellite cells.^5,36,47,52^ This problem hampers the application of satellite cell in clinic. Our results revealed that pig satellite cells have the same problem. It suggests that the factors required for stemness maintenance are lacking in the *in vitro* culturing system. Recently, Fu *et al.* found that cytokines secreted by activated T cells during the acute inflammation after muscle injury can help maintain the stemness of mouse satellite cells *in vitro* for over 2 months.^36^ Charville *et al.*^47^ showed that inhibition of p38 signaling is important to maintain the quiescent stage and stemness of human satellite cells. Whether these factors also work to maintain stemness of pig satellite cells is an interesting question to pursue.

## Materials and Methods

### Animals and pig muscle tissues

All animal procedures were performed according to the institutional and national guidelines and approved by the Shanghai Institute of Biochemistry and Cell Biology, Chinese Academy of Sciences in this study. Pig muscle tissues were collected from commercial pig ‘large white’ at the time of killing (asphyxiated by CO_2_ of 1 week old, or stunned electrically and then killed by cutting the jugular vein of 25 week old). The NOD.Cg-*Prkdc*^*scid*^ *Il2rg*^*tm1Wjl*^/SzJ (NSG) mice were purchased from Jackson Laboratories (Bar Harbor, ME, USA).

### Pig satellite cells isolation

Pig satellite cells were isolated from 1- or 25-week-old male pigs as previously described and adapted to pig tissues.^26,47^ Briefly, freshly collected pig muscle was either immediately digested or kept in DMEM (Gibco, Grand Island, NY, USA) at 4 °C. The tissues were dissected and dissociated with collagenase D (Roche 2 mg/ml, Indianapolis, IN, USA) and dispase II (Roche 1.07 U/ml) in DMEM supplement with 1% penicillin-streptomycin (P.S.) at 37 °C for 1.5 h. The mixture was triturated with pipette once per 15–20 min. After digestion, the muscle was aspirated and ejected in and out of the syringe 10 times with a 20-ml syringe and a 20-gauge needle. After centrifuging at 100×*g* for 5 min, the supernatant was collected and centrifuged at 1000×*g* for 5 min at 4 °C. The cells were washed with 20% FBS in DMEM and filtered through a 100 *μ*m cell strainer followed by a 40 *μ*m cell strainer. The cells were then centrifuged at 1000×*g* for 5 min at 4 °C and incubated with the erythrocyte lysis buffer (ACK) buffer for 5 min on ice. Then the cells were washed with PBS twice and cell pellet was reconstituted with FACS buffer (1% BSA in PBS) or frozen in FBS supplement with 10% dimethyl sulfoxide (DMSO).

For freezing cells, the cells were recovered in 37 °C water bath and washed with PBS twice. The cells were reconstituted with FACS buffer and stained with an antibody cocktail consisting Alexa Fluor 647 anti-pig CD45 (1 : 20 BIO-RAD, Richmond, CA, USA; Cat# MCA1222A647), APC-conjugated anti-pig CD31 (1 : 20 BIO-RAD, Cat# MCA1746APC), Alexa Fluor 488 anti-human CD29 (1 : 40 BioLegend, San Diego, CA, USA; Cat# 303016), PE-conjugated anti-human CD56 (1 : 40 BioLegend; Cat# 304606) for 30–45 min on ice. After antibody incubation, the cells were washed with cold PBS for two times and reconstituted in F-10 with 20% FBS. The viable CD31^−^CD45^−^CD56^+^CD29^+^ cells were isolated. Also the CD31^+^CD45^+^ cells and CD31^−^CD45^−^CD29^+^CD56^−^ cells were also isolated to extract RNA. Cell sorting was performed with a BD Influx cell sorter using 488, 561, and 640 nm lasers. Unstained cells were routinely used to define FACS gating parameters.

### Satellite cell culture and differentiation

Dishes (Corning, Corning, NY, USA) were coated with 0.05% collagen type I (Corning). FACS isolated pig satellite cells were cultured on collagen-coated dishes in F10 medium (F10 medium (Gibco) containing 15% FBS, 5 ng/ml FGF (Invitrogen, Carlsbad, CA, USA) and 1% P.S.). Freshly isolated satellite cells were collected and plated in collagen-coated dish and cultured for less than 6 h to attach. Cells cultured at various hours were fixed with 4% paraformaldehyde (PFA) for immunofluorescent staining. For serial expansion, cells were serially passaged to maintain a density of <60% confluence and counted at each passage. Pig satellite cells differentiation were induced with DMEM (Invitrogen) with 2% horse serum (Hyclone, Logan, UT, USA) or 0.4% Ultroser G (Pall Corporation 15950-017; East Hills, NY, USA). Differentiation was induced for 4 days. The expanded and differentiated cells were fixed with 4% PFA for immunofluorescent staining.

To observe the myotube contraction, pig satellite cells were differentiated in DMEM containing 0.4% Ultroser G (Pall Corporation 15950-017) for 6 days, medium was changed at day 4.

For preplating method, isolated mononuclear cells were firstly cultured in F10 basal medium for 2 days. Cells were then treated with 0.25% trypsin for 5 min and stopped with F10 basal medium, centrifuged and re-plated. To separate satellite cells from fibroblast cells, the cells were incubated in an uncoated dish at 37 °C for 60 min and then transferred to a new collagen-coated dish. This preplate technical was repeated three times to remove residual fibroblast cells. The bright field images were acquired by Olympus DP73 microscope.

### Immunofluorescent analysis of cultured cells

Cultured satellite cells were fixed with ice-cold 4% PFA (in PBS) for 20 min, rinsed with PBS, and permeabilized in 0.5% Triton X-100 (in PBS) for 8 min. Fixed and permeabilized cells were blocked and incubated with primary antibodies 1% BSA (in PBS) overnight at 4 °C. Primary antibodies recognizing Pax7 (1 : 100, Developmental Studies Hybridoma Bank, Iowa City, IA, USA;, Shanghai, China; Cat# PAX7), MyoD1 (1 : 200, abclonal, Shanghai, China; Cat# A0671), myosin heavy chain (1 : 1000, Upstate, Temecula, NY, USA; Cat# 05–716). After washing with PBS, cells were incubated with secondary antibodies for 1 h at room temperature. Next, the cells were stained with Alexa 488-, 561-labeled anti-mouse or -rabbit antibodies (Invitrogen) and then mounted with VECTASHIELD mounting medium with DAPI (Vector Laboratories, Burlingame, CA, USA, Cat# H-1200). All images were acquired by Leica SP8 confocal microscope and processed with Adobe Photoshop CS5 to adjust brightness and contrast for publication.

For the FACS analysis of cultured satellite cells, about 10^5^ cells were collected at each passage. The cells were fixed by 2% PFA for 15 min and washed for 5 min three times. The cells were then incubated with Alexa Fluor 488 anti-human CD29 (1 : 40 BioLegend, Cat#303016), PE-conjugated anti-human CD56 (1 : 40 BioLegend, Cat# 304606) in 1% BSA in PBS for 30–45 min. After antibody incubation, the cells were washed with PBS for two times and reconstituted in PBS. FACS analysis was performed in LSR II (BD Biosciences, San Jose, CA, USA) cell analyzer. Unstained cells were routinely used to define FACS gating parameters.

### Pig muscle analysis

The pig muscle tissues were cut into small pieces and then frozen in liquid nitrogen. Cross sections were cut at 8 *μ*m and processed for immunofluorescent staining. The cross sections were fixed with 4% PFA at room temperature for 10 min, washed in PBS. The sections were permeabilized with 0.5% Triton X-100 for 6 min and washed with PBS again. Sections were then blocked for 1 h with 10% goat serum at room temperature. For Pax7 and Laminin co-staining, sections were incubated at 4 °C overnight with the following primary antibodies: anti-Laminin antibody (1 : 50, Sigma, St Louis, MO, USA; Cat# L9393) or rat anti-Laminin (1 : 1000, Abcam, Cambridge, MA, USA; Cat# 11576), mouse anti-Pax7 (1 : 25, Developmental Studies Hybridoma Bank, Cat# PAX7). After PBS wash for 5 min three times, the sections were stained with Alexa 488-, 561- or 647-labeled anti-mouse or -rabbit or -rat antibodies (Invitrogen) and then mounted with VECTASHIELD mounting medium with DAPI (Vector Laboratories, Cat# H-1200).

For sections co-stained with Pax7, Laminin, and CD56/CD29, Pax7 and Laminin co-staining were first performed. After incubation with the secondary antibody above, sections were washed with PBS for 5 min three times and incubated with the Alexa Fluor 488 anti-human CD29 (1 : 25, BioLegend, Cat# 303016), APC anti-human CD56 (1 : 25, BioLegend, Cat# 304610) for 2–4 h at room temperature. After washing with PBS for three times, sections were mounted with VECTASHIELD mounting medium with DAPI (Vector Laboratories, Cat# H-1200). All images were acquired by Leica SP8 confocal microscope and processed with Adobe Photoshop CS5 to adjust brightness and contrast for publication.

### Pig satellite cells transplantation

NSG mice were pretreated with 18 gamma (Gy) 1 day before transplantation limited to the hindlimb. Muscle injury was induced by the injection of CTX (Sigma) to TA muscle. Briefly, 15 *μ*l of 10 *μ*M CTX was injected at each TA muscle using 28 gauge needles. After 24 h, freshly isolated and cultured pig satellite cells (treated by trypsin) at passage 2 and 5 were counted, centrifuged, and resuspended in 15 *μ*l PBS with 10^5^ cells. Cells were injected into the TA muscle using 28 gauge needles. Recipient mice were treated with daily drinking water supplement with neomycin sulfate (0.25 mg/mg) for 2 weeks. The transplanted TA muscles were collected 1 week, 4 weeks after transplantation in liquid nitrogen. Serial 10 *μ*m transverse sections of the TA muscle were stored in −80 °C fridge.

All glass slides were removed from −80 °C and warmed at room temperature for 10 min. Sections were rehydrated with PBS and fixed in 4% PFA for 10 min at room temperature. After washed with PBS for 5 min three times, the sections were permeabilized with 0.5% Triton X-100 for 6 min and again washed with PBS. Then sections were blocked for 1 h in 10% goat serum (in PBS) with mouse-on-mouse blocking reagent (Vector Laboratories). After blocking, sections were washed with PBS and incubated overnight with primary antibodies at 4 °C. Next day, sections were then washed with PBS for 5 min three times and incubated with secondary antibodies 1 h at room temperature. Finally, sections were washed with PBS and mounted with VECTASHIELD mounting medium with DAPI (H-1200 Vector Laboratories). Antibodies used in the staining were mouse anti-Pax7 (1 : 20, Developmental Studies Hybridoma Bank, Cat# PAX7), mouse anti-Myh3 (1 : 100, Developmental Studies Hybridoma Bank), mouse anti-Lamin A/C (1 : 50, Santa Cruz Biotechnology, Santa Cruz, CA, USA; Cat# sc-7292), rabbit anti-Dystrophin antibody (1 : 50, Abcam, Cat# ab15277). The sections were next stained with Alexa 488-, 561-labeled anti-mouse or -rabbit antibodies (Invitrogen). All images were acquired by Leica SP8 confocal microscope and processed with Adobe Photoshop CS5 to adjust brightness and contrast for publication.

### Gene expression analysis

RNA was extracted from cells using the RNAprep pure cell/bacteria kit including DNase treatment (TIANGEN, Beijing, China) according to the manufacturer's instruction. One microgram of total RNA from each sample was reverse transcribed to cDNA using M-MLV transcriptase (Promega, Madison, WI, USA) according to the manufacturer’s instruction. Relative gene expression was determined and performed in triplicate using a SYBR Green PCR master mix on an ABI 7500 fast real-time PCR system.

The primers used in these assays were the followings: *Pax7*-F, 5′- GGTGGGGTcTTCATCAATGG-3′, *Pax7*-R, 5′- GTCTCTTGGTAGCGGCAGAG-3′, *Myf5*-F, 5′- AGAAGGTCAACCAGGCGTTT-3′, *Myf5*-R, 5′- GTAGCGGATGGCATTCCTGA-3′, *GAPDH*-F, 5′- CAAGGAGTAAGAGCCCCTGG-3′, *GAPDH*-R, 5′- AGTCAGGAGATGCTCGGTGT-3′.

### 5′ and 3′ RACEs

RNA was isolated from primary pig myoblasts by preplate method. The original sequence was based on the published partial pig *Pax7* cDNA sequence (Accession #: AY653213). We designed four pairs of gene-specific primers: P1, P2, P3, and P4 for 5′-RACE, P5, P6, P7, and P8 for 3′-RACE. RACE experiments were performed with SMARTer RACE 5′/3′ Kit (Clontech, San Jose, CA, USA) following the manufacturers’ protocols. Finally, we cloned the result cDNA into the pUC19-based vector and sequenced. The peptide and nucleotide sequences were aligned by the NCBI online Basic Local Alignment Search Tool.

The primers used were: P1, 5′- GGTGGGGTTTTCATCAATGGGCGACC-3′, P2, 5′- CCACATCCGCCACAAGATAGTAGAG-3′, P3, 5′- TGCCCAACCACATCCGCCACAAGATAG-3′, P4, 5′- AGTAGAGATGGCCCACCACGGCATCCG-3′, P5, 5′- GTCTCTTGGTAGCGGCAGAGGAT-3′, P6, 5′- AGGCCGGATGGACCCGGTCTCTTGG-3′, P7, 5′- TGTCTGGGCTTGCTGCCTCCAATGG-3′, P8, 5′- TCCACATCCGGAGTCGCCACCTGTCTG-3′.

### Cell size, Pax7^+^ cells population, differentiation, and engraftment efficiency measurement

The cell sizes were measured by ImageJ software (NIH). Bright field images of satellite cells cultured at different time points were acquired using Olympus DP73 microscope. Immediately after isolation, freshly isolated satellite cells were first plated and allowed to adhere for less than 6 h at 37 °C.

Pax7^+^ cells population was expressed as the number of nuclei co-stained with DAPI and Pax7 divided by the total number of nuclei in the same field. More than 400 nuclei from five randomly chosen fields were analyzed.

MyHC^+^ cells population was expressed as the number of nuclei co-stained with MyHC^+^ and DAPI divided by the total number of nuclei in the same field. More than 3000 nuclei from four randomly chosen fields were analyzed.

The percentage of myotube nuclei in total nuclei was expressed as the nuclei inside myotube (at least three nuclei) divided by total nuclei in the same field. More than 3000 nuclei from four randomly chosen fields were analyzed.

### Statistics

Statistical analyses were performed using GraphPad Prism 5 (GraphPad Software). For comparisons of two treatment groups, a student’s *t*-test was used. Results were means±S.E.M. unless stated. *P*<0.05 was considered significant.
