# Supplementary material for: Characterization and isolation of highly purified porcine satellite cells
Source: Cell Death Discov. 2017 Apr 10;3:17003–. doi: 10.1038/cddiscovery.2017.3 (PMC5385392; doi:10.1038/cddiscovery.2017.3)
Supplement: Supplementary Information [file cddiscovery20173-s1.docx]

**Online Supplemental Information**

**Figure S1. Related to Figure 1.** **Comparison of the partial peptide sequences** **of Pax3 and Pax7 in cattle, human, mouse and pig.**

The comparison of partial predicted peptide sequences of Pax3 and Pax7 in cattle, human, mouse and pig. The green and blue background indicated differences on two key amino acids between Pax3 and Pax7 peptide sequence.

**Figure S2. Related to Figure 1.** **Identification of the completed cDNA** **sequence of pig Pax7 by RACE.**

(a) The strategy to generate full-length pig Pax7 cDNA sequence by RACE. The red band indicated the seed sequence for RACE. (b) The 5’ and 3’ cDNA fragments generated by 5’ and 3’ RACE reactions. GSP indicated gene specific primer, NGSP indicated nested gene specific primer. The band generated by 5’ RACE was about 400bp (b left) and a 5kb fragment was generated by the 3’RACE (b right).

**Figure S3. Related to Figure 1.** **Alignment of** **pig Pax7 polypeptide sequence from different species.**

(a) Alignment of the full length pig Pax7 CDS with human and mouse Pax7. Shadow indicated the conserved domains. Human (*Homo sapiens*, Hs) and mouse (*Mus musculus*, Ms) Pax7 cDNA sequences were aligned with assembled pig Pax7 cDNA sequence. (b) The human and mouse Pax7 polypeptide sequences were aligned with a pig Pax7 polypeptide sequence derived from full length CDS.

**Figure S4. Related to Figure 3. Identification of the cell surface marker for swine satellite cells.**

(a) RT-qPCR analysis of Myf5 mRNA levels in freshly isolated pig satellite cells and other cell populations. Error bars represented S.E.M. and were based on 4 independent experiments. Significance was analyzed by student’s t-test, *** indicated p < 0.001. (b) Immunofluorescent staining of Pax7 in pig satellite cells cultured 4 days in vitro. Cells were stained with antibodies against Pax7 and with DAPI to identify nuclei. Blue indicated DAPI; green indicated Pax7. Scale bars: 50 μm.

**Figure S5. Related to Figure 4.** **In vitro proliferation and differentiation features of pig satellite cells.**

(a) Representative phase contrast images of pig satellite cells cultured in F-10 medium for various hours. Scale bars: 100 μm. (b) Representative phase-contrast pictures of 2x10^4^, 1x10^5^, 5x10^5^ cells cultured in F10 medium in 3.5cm dish for 2 days. Scale bars: 200 μm.

**Figure S6. Related to Figure 6.** **Swine satellite cells contributed to muscle regeneration in recipient mice.**

(a) Four weeks after transplantation, engraftment efficiency of freshly isolated pig satellite cells was determined by Dystrophin and Lamin A/C immunofluorescent staining with muscle sections derived from receipient mice. PBS was injected as negative control. Blue indicated DAPI; red indicated Lamin A/C; green indicated Dystrophin. Scale bars: 100μm. (b) Pax7, immunofluorescent staining with muscle sections derived from recipient mice four weeks after transplantation. Blue indicated DAPI; red indicated Pax7; purple indicated Dystrophin. Arrow and triangle indicated Pax7 expressing satellite cells. Scale bars: 100μm. (c) Pax7, Lamin A/C immunofluorescent staining with series muscle sections derived from recipient mice four weeks after transplantation. Blue indicated DAPI; red indicated Pax7 (c, top) or Lamin A/C (c, bottom); purple indicated Dystrophin. Scale bars: 20μm.

**Supplementary file S7.** **Myotubes differentiated from swine satellite cells were capable of contraction.**

Differentiated pig satellite cells were maintained in UG for 6 days. Myotube contraction could be observed.
