# Supplementary material for: Characterization and isolation of highly purified porcine satellite cells
Source: Cell Death Discov. 2017 Apr 10;3:17003–. doi: 10.1038/cddiscovery.2017.3 (PMC5385392; doi:10.1038/cddiscovery.2017.3)
Supplement: Supplementary Figure S1 [file cddiscovery20173-s2.pdf]

Ding S et al. Supplementary Figure S1. Related to Figure 1

**Pax3(Bt)**	1	GGVFINGRPLPNHIRHKIVEMAHHGIRPCVISRQLRVSHGCV	42
**Pax3(Hs)**	1	GGVFINGRPLPNHIRHKIVEMAHHGIRPCVISRQLRVSHGCV	42
**Pax3(Ms)**	1	GGVFINGRPLPNHIRHKIVEMAHHGIRPCVISRQLRVSHGCV	42
**Pax3(Ss)**	1	GGVFINGRPLPNHIRHKIVEMAHHGIRPCVISRQLRVSHGCV	42
**Pax7(Bt)**	1	GGVFINGRPLPNHIRHKIVEMAHHGIRPCVISRQLRVSHGCV	42
**Pax7(Hs)**	1	GGVFINGRPLPNHIRHKIVEMAHHGIRPCVISRQLRVSHGCV	42
**Pax7(Ms)**	1	GGVFINGRPLPNHIRHKIVEMAHHGIRPCVISRQLRVSHGCV	42
**Pax7(Ss:AAT72072)**	1	GGVFINGRPLPNHIRHKIVEMAHHGIRPCVISRQLRVSHGCV	42
**Consensus**	1	GGVFINGRPLPNHIRHKIVEMAHHGIRPCVISRQLRVSHGCV	42

**Pax3(Bt)**	43	SKILCRYQETGSIRPGAIGGSKPKQVTTPDVEKK	76
**Pax3(Hs)**	43	SKILCRYQETGSIRPGAIGGSKPK–VTTPDVEKK	76
**Pax3(Ms)**	43	SKILCRYQETGSIRPGAIGGSKPKQVTTPDVEKK	76
**Pax3(Ss)**	43	SKILCRYQETGSIRPGAIGGSKPKQVTTPDVEKK	76
**Pax7(Bt)**	43	SKILCRYQETGSIRPGAIGGSKPR–VATPDVEKK	76
**Pax7(Hs)**	43	SKILCRYQETGSIRPGAIGGSKPRQVATPDVEKK	76
**Pax7(Ms)**	43	SKILCRYQETGSIRPGAIGGSKPRQVATPDVEKK	76
**Pax7(Ss:AAT72072)**	43	SKILCRYQETGSIRPGAIGGSKPRQVATPDVEKK	76
**Consensus**	43	SKILCRYQETGSIRPGAIGGSKP QV TPDVEKK	76
