# Supplementary figures and images for: Characterization and isolation of highly purified porcine satellite cells
Source: Cell Death Discov. 2017 Apr 10;3:17003–. doi: 10.1038/cddiscovery.2017.3 (PMC5385392; doi:10.1038/cddiscovery.2017.3)

Ding S et al. Supplementary Figure S2. Related to Figure 1

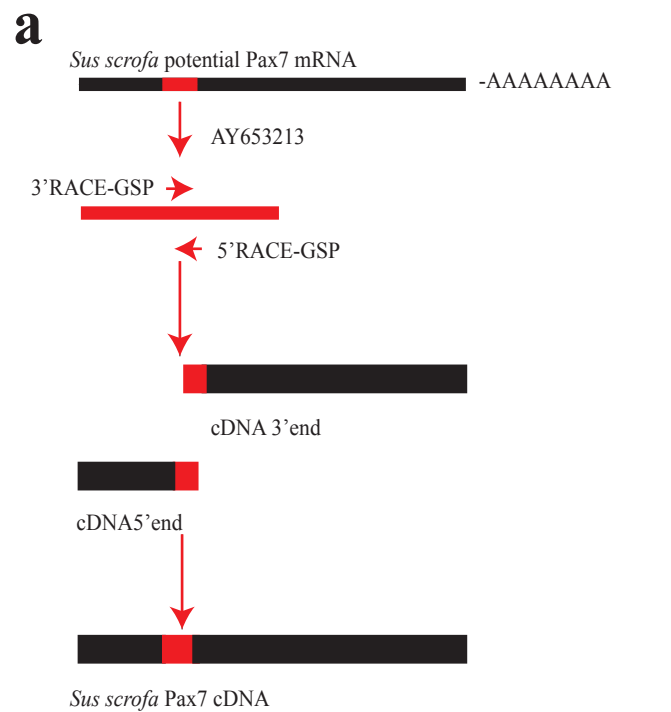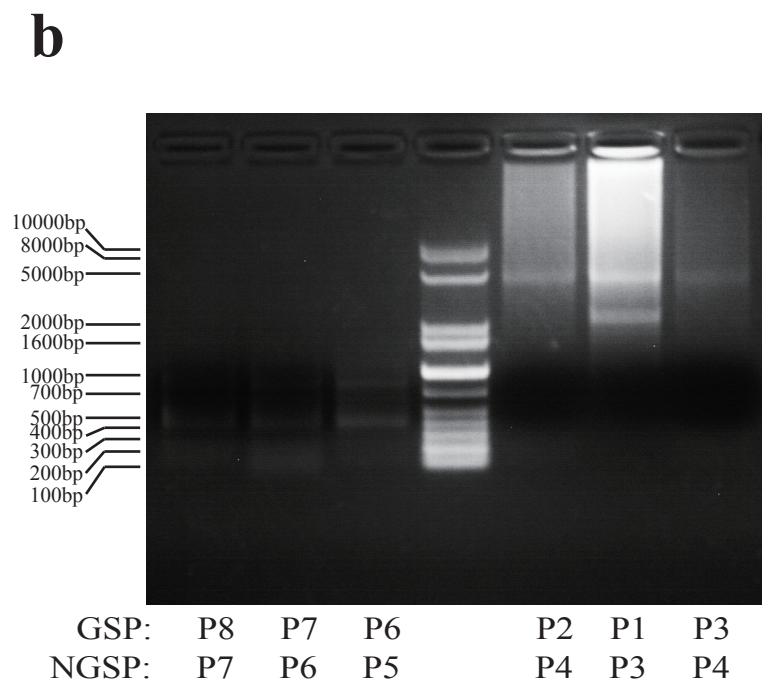

Supplement: Supplementary Figure S2 [file cddiscovery20173-s3.pdf]

# Ding S et al. Supplementary Figure S3. Related to Figure 1

**a**

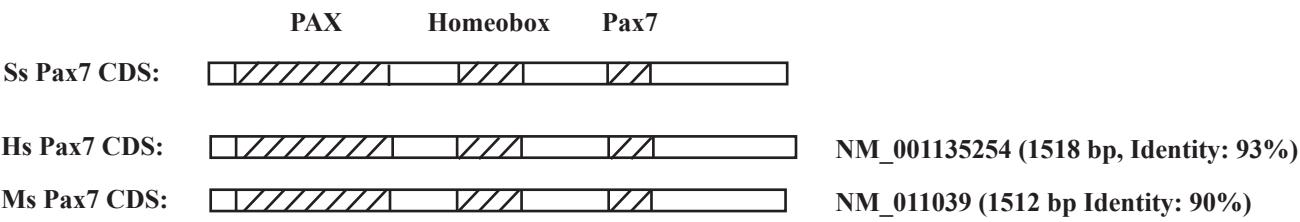

**b**

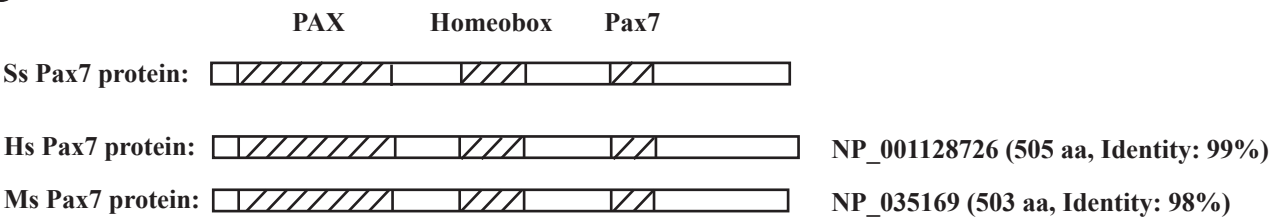

Supplement: Supplementary Figure S3 [file cddiscovery20173-s4.pdf]

# Ding S et al. Supplementary Figure S4. Related to Figure 3

**a**

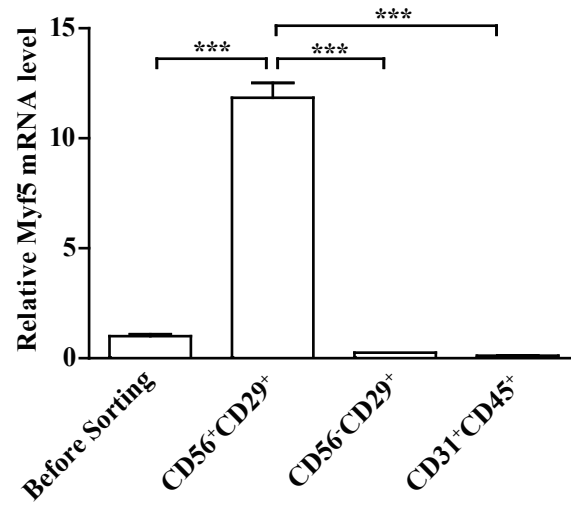

**b**

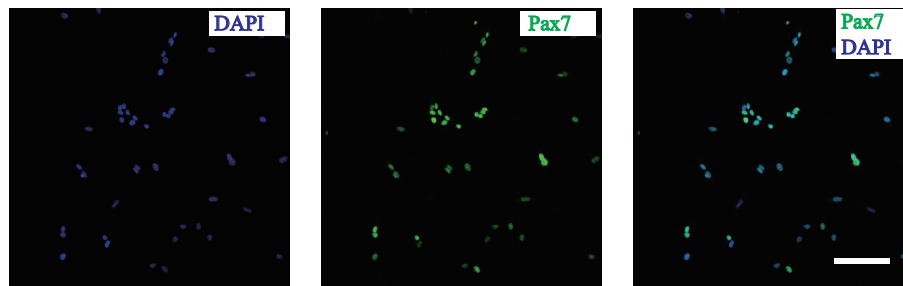

Supplement: Supplementary Figure S4 [file cddiscovery20173-s5.pdf]

# Ding S et al. Supplementary Figure S5. Related to Figure 4

**a**

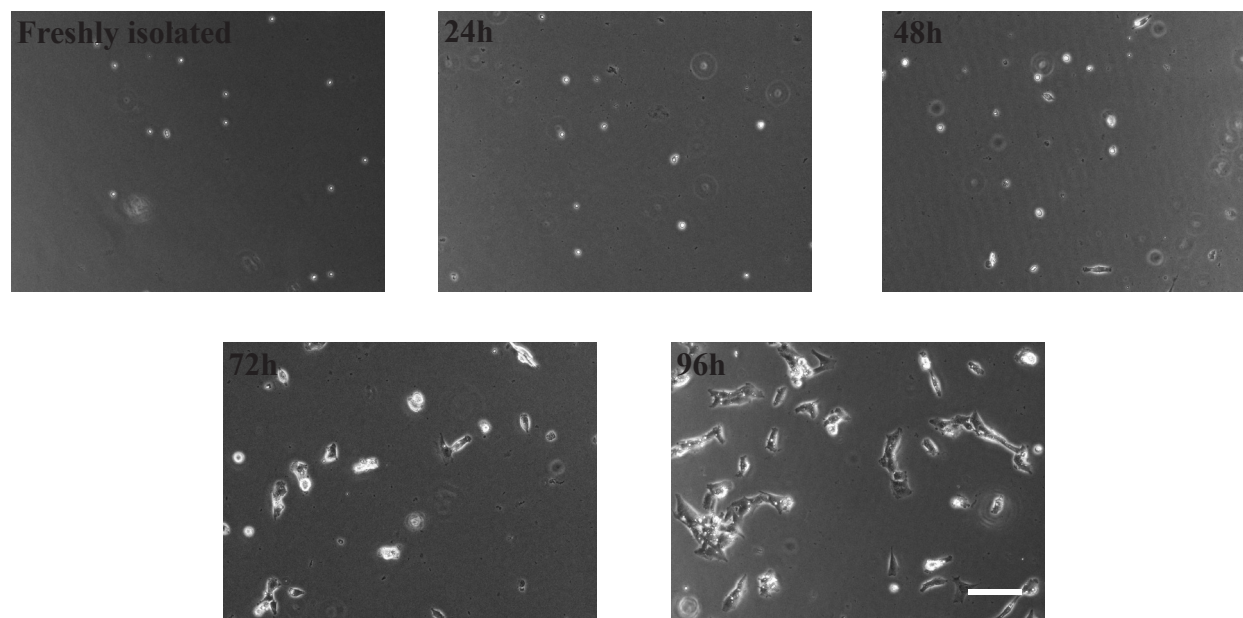

**b**

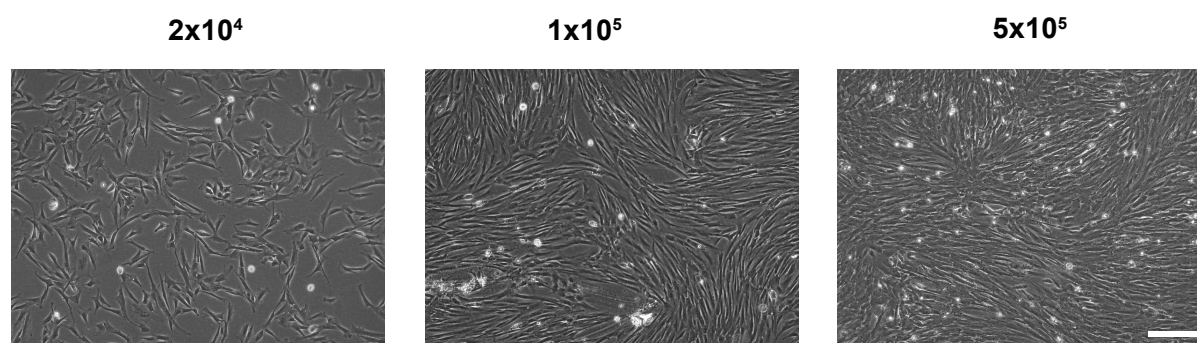

Supplement: Supplementary Figure S5 [file cddiscovery20173-s6.pdf]

# Ding S et al. Supplementary Figure S6. Related to Figure 6

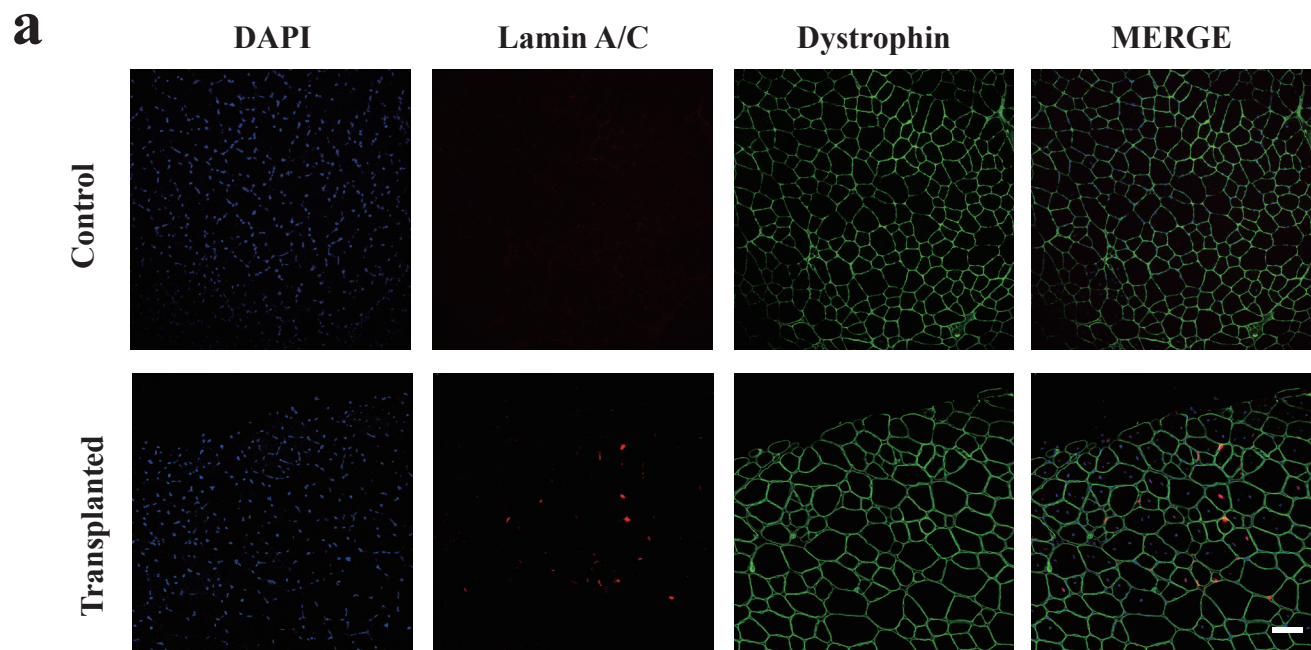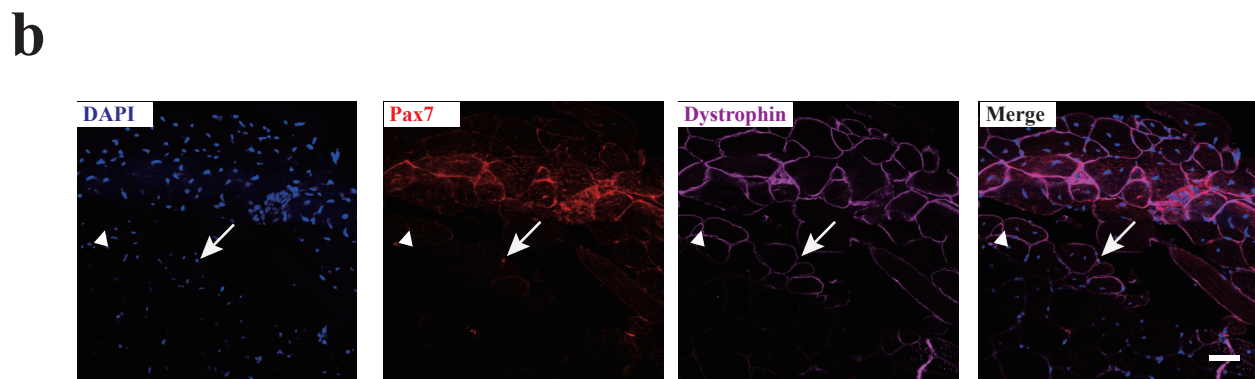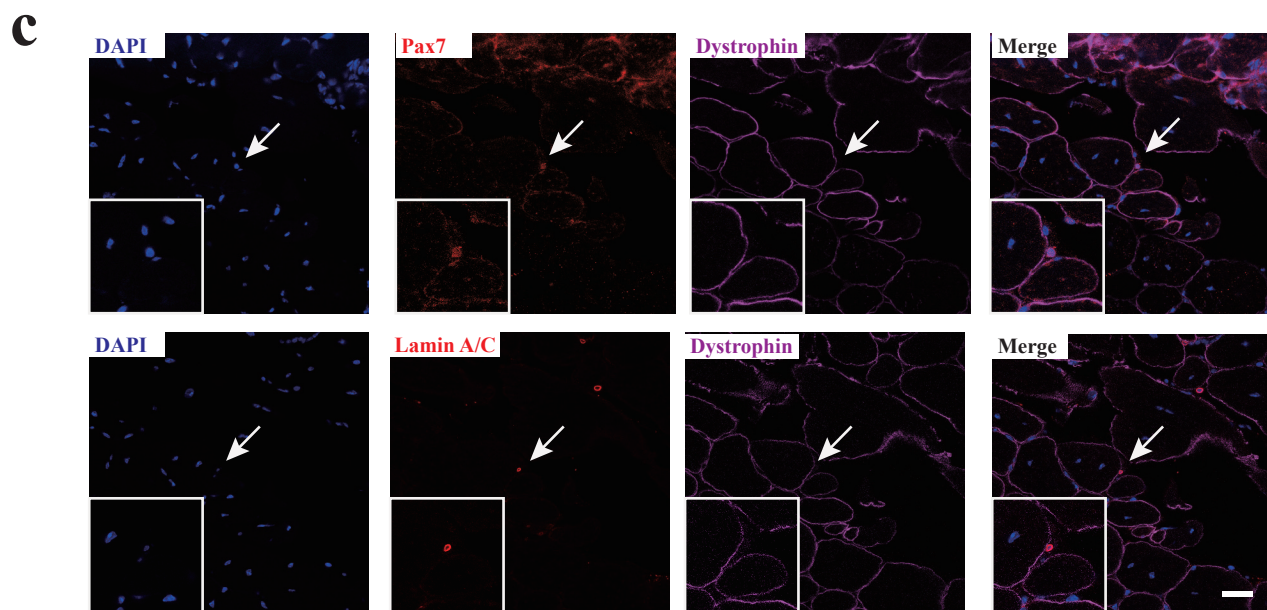

Supplement: Supplementary Figure S6 [file cddiscovery20173-s7.pdf]
